# Erdheim-Chester disease presenting as meningitis with hypoglycorrhachia: A case report

**DOI:** 10.1097/MD.0000000000030585

**Published:** 2022-09-16

**Authors:** Christopher Polk, Carol Weida, Nikhil Patel, Michael Leonard

**Affiliations:** a Division of Infectious Diseases, Atrium Health, Charlotte, NC; b Department of Pathology, Atrium Health, Charlotte, NC; c Division of Pulmonary and Critical Care, Atrium Health, Charlotte, NC.

**Keywords:** Erdheim-Chester disease, meningitis, hypoglychorrhachia

## Abstract

Erdheim-Chester disease (ECD) commonly has neurologic manifestations but rarely presents with meningitis and hypoglycorrhachia. Here, were present a case of ECD with a clinical and laboratory presentation initially thought to be bacterial meningitis with sepsis.

**Methods:**

We report a case of a 79-year-old with history of enigmatic bone pain and peritoneal nodules who presented with meningitis. After failure to improve on antibiotic therapy other etiologies of hypoglycorrhachia including sarcoid, tuberculosis, and fungal and carcinomatous meningitis were considered. However, no definite diagnosis could be made based on radiologic, serologic, microbiologic, and molecular testing and the patient failed to improve on empiric therapy including antibiotics, antifungals, and tuberculosis and steroid therapy.

**Results:**

Ultimately, autopsy revealed a new diagnosis of ECD manifesting as meningitis, a rare presentation of a rare disease.

**Conclusion:**

Although only reported in one other case to our knowledge, ECD can present with meningitis with hypoglycorrhachia.

## 1. Introduction

Hypoglycorrhachia traditionally is attributed to a limited number of etiologies: bacterial or fungal meningitis, tuberculosis, neoplasm, sarcoid, or lupus cerebritis.^[1]^ Infectious disease physicians may consider this differential diagnosis in approaching cases with hypoglycorrhachia, especially those initially labeled as bacterial meningitis without definitive microbiologic confirmation and failure to improve on antibiotic therapy. The patient’s prior medical history may significantly impact degree of suspicion for each of these diagnoses, particularly carcinomatous meningitis which is usually a later presentation of a known malignancy, most commonly lymphoma.^[2]^ Here, we describe, with permission from the patient’s medical decision makers and under institutional review board exemption, an initial presentation of a rare malignancy, Erdheim-Chester disease (ECD) presenting as meningitis.

## 2. Case

A 79 year old, retired internal medicine physician presented with 1 month of bilateral posterior auricular headaches and 3 days of fevers and diffuse weakness. He had undergone ventral abdominal hernia repair 1 month prior and was treated with amoxicillin/clavulanic acid and doxycycline for pneumonia just prior to initiation of headaches. His medical history was also significant for Crohn disease treated with oral budesonide with a negative Quantiferon Gold test sent 9 months prior, a seronegative rheumatoid arthritis diagnosis within the past year due to worsening joint and leg pain, and migraine headaches. Retrospective chart review was notable for finding a fibrinous exudate material on the liver, intestines, omentum, and in his pelvis during his recent ventral hernia repair, which were similar to findings noted during an appendectomy 4 years prior. Biopsy of a peritoneal implant during surgery revealed mesothelial line fibrous tissue with acute inflammation but was read as negative for malignancy and acid fast bacilli cultures sent from appendectomy surgery 4 years ago were without growth.

On hospital admission, he had a leukocytosis of 13,000/μL but negative respiratory pathogen panel and SARS-CoV-2 nasopharyngeal swab, bland urinalysis, and blood and urine cultures were without growth. However, renal ultrasound demonstrated bilateral perinephric fluid, so he was treated for a presumed urinary tract infection with cefepime. On hospital day 5, he became confused and nonverbal prompting a lumbar puncture with cerebrospinal fluid (CSF) revealing a neutrophilic meningitis with hypoglycorrhachia (see Table 1). CSF gram stain and cultures, cryptococcal antigen, and Herpes simplex virus 1/2 and Varicella Zoster virus polymerase chair reactions (PCRs) were negative. Magnetic resonance imaging brain obtained revealed trace material in the right greater than left occipital horns demonstrating diffusion restriction and mild focal ependymal enhancement concerning for ventriculitis. Treatment was changed to dexamethasone, vancomycin, ceftriaxone, and ampicillin.

**Table 1:** CSF analysis over time.

	Day 5	Day 12	Day 21
**WBC/μL**	840 neutrophils 75%	194 monocytes 87%	32 neutrophils 50%
**RBC/μL**	250	1	13
**Glucose mg/dL**	<10	<10	<10
**Protein mg/dL**	190	118	93
**Current treatment** *	Cefepime	Dexamethasone, Vancomycin, Ceftriaxone, Ampicillin	Dexamethasone, Vancomycin, Meropenem, RIPE†

* at time lumbar puncture performed.

† Rifampin, Isoniazid, Pyrazinamide, Ethambutol.

CSF = cerebrospinal fluid.

On hospital day 12, given a lack of clinical improvement repeat lumbar puncture was performed again revealing a meningitis, now with monocytic predominance with hypoglycorrhachia (see Table 1). Repeat magnetic resonance imaging revealed persistent diffusion restriction of the occipital horns consistent with ventriculitis. Further imaging with computed tomography (CT) Chest was only notable for small bilateral pleural effusions and positron emission tomography (PET)-CT noted increased update in the left lower lobe of the lung consistent with pneumonia, and increased update in the spleen with splenomegaly. Clinically, he remained confused and developed systemic hypotension requiring ICU transfer and vasopressor therapy. Antibiotics were switched to vancomycin and meropenem, and empiric treatment of tuberculosis (TB) begun with rifampin, isoniazid, pyrazinamide, and ethambutol. He remained on steroids. Despite this another repeat lumbar puncture obtained on hospital day 19 again revealed persistent hypoglycorrhachia with neutrophilic meningitis and he clinically remained unimproved (see Table 1). CSF studies sent from his 3 lumbar punctures in total returned with 2 negative cryptococcal antigens, TB PCR negative twice, and negative Venereal Disease Research Laboratory test. Herpes simplex virus 1 and 2 PCR, Varicella Zoster virus PCR, John Cunningham virus PCR, Enterovirus PCR, bacterial, fungal, and acid fast bacilli cultures negative and fluid cytology negative for malignancy. An elevated CSF angiotensin converting enzyme level was noted at >11.9 U/L. Due to his continue clinical worsening Liposomal Amphotericin was added, but on hospital day 21 family elected to withdraw care due to his continued clinical worsening and shock. An autopsy was performed.

**Figure 1.**
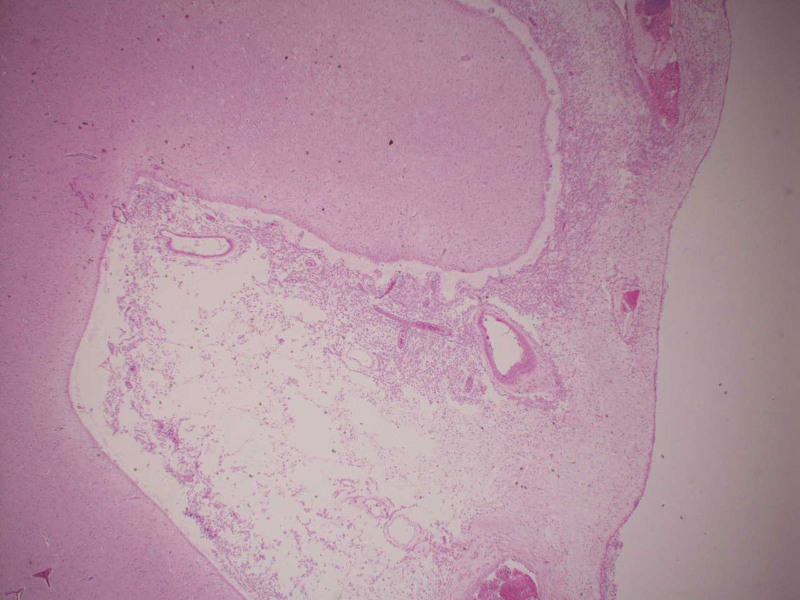
20×, Hematoxylin & Eosin, Thick meninges with infiltrative histiocytic cells.

**Figure 2.**
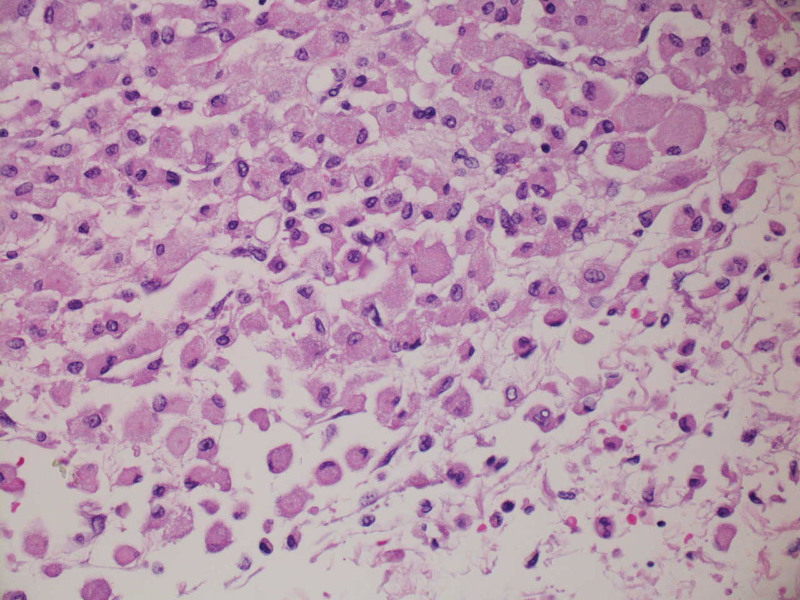
400×, Hematoxylin & Eosin. High power image of meninges showing the morphology of the histiocytes: large cells with eccentric nuclei and abundant granular cytoplasm.

**Figure 3.**
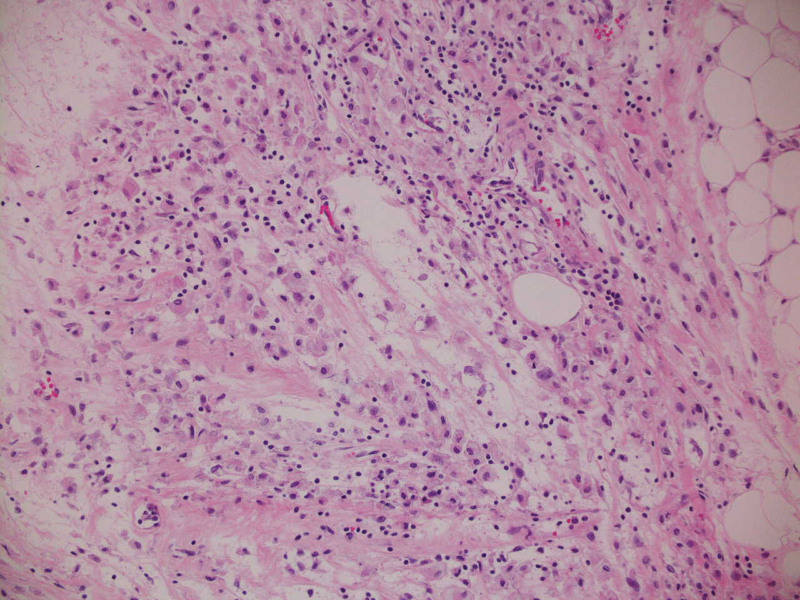
200×, Hematoxylin & Eosin. Perisplenic tissue demonstrating similar histiocytic infiltrate.

At autopsy, the meninges were thick and gelatinous (see Fig. 1). Corresponding histologic sections demonstrated an infiltrative cellular process with an associated mixed inflammatory population consisting of neutrophils, eosinophils, and lymphocytes. The initial impression was meningeal carcinomatosis. An immunohistochemical stain for pancytokeratin was performed and was negative, ruling out carcinoma. In addition, no malignancy was identified in any organ system. Additional evaluation revealed that the infiltrative cells stained with immunohistochemical macrophage marker CD68 (see Fig. 2). Similar cells were seen expanding the right choroid plexus and infiltrating the spinal cord meninges. Immunohistochemical stains for CD1a and BRAF were negative as was the molecular probe for BRAF. Additional autopsy findings included a histiocytic infiltrate in the perirenal fat, omentum, spleen, and perihilar lymph nodes (see Fig. 3). A histiocytic infiltrate was also seen on the surface of the gallbladder. In the perirenal fat, the histiocytic infiltrate was inciting a fibrous response. The negative CD1a stain essentially ruled out a Langerhans histiocytosis. Diagnosis was made of ECD.

## 3. Discussion

ECD is a non-Langerhan histocyte neoplasm first described in 1930 by Jakob Erdheim and William Chester.^[3,4]^ The World Health Organization classified ECD as a distinct entity in 2016, separate from disseminated juvenile xanthogranoloma, characterized by CD68-positive, CD1a-negative foamy histocytes infiltrating into tissue which can occur in multiple organs.^[3,5,6]^ It is associated with mutations in the mitogen-activating protein kinase cell signaling pathway involving RAS-RAF-MEK-ERK, with patients in 1 case series of neurologic ECD found harboring mutations in BRAF (18/30 patients), MAP2K1 (5/30 patients), RAS isoforms (2/30 patients), or fusions of BRAF and ALK (2/30 patients).^[7]^

The disease presents more often in males.^[4,7]^ While often involving multiple organ systems, almost all patients with ECD have some bone involvement on imaging, with bone pain being clinically apparent in 50% of patients over the course of their illness.^[2,5]^ The bone pain usually involves the knees or leg as the femur, tibia, and fibula are most commonly involved, as in our patient, with the axial skeleton usually spared.^[2]^ ECD may also effect the pulmonary and cardiovascular system with lung fibrosis, coronary vessel infiltration and congestive heart failure, and periaortic fibrosis.^[4]^ A thickened pericardium and pericardial effusion may also result.^[4]^ Pulmonary and cardiovascular involvement along with neurologic involvement as in our patient portend a worse prognosis.^[5]^ Up to one-third of patients may have neurologic involvement with about 23% presenting with neurologic symptoms.^[5,8]^ Diabetes insipidus may result from pituitary involvement.^[5]^ Interestingly patients present with neurologic involvement on average in their late 40s while constitutional symptoms and retroperitoneal fibrosis are more common presentations in older patients with ECD.^[5]^ Constitutional symptoms can consist of fevers, night sweats, fatigue, weakness, and weight loss.^[4]^ Our patient had bone and peritoneal involvement which predated by years his development of constitutional and neurologic symptoms, which is not uncommon. A delay in diagnosis of 2–5 years is described in multiple case series of patients with neurologic ECD.^[7,8]^ Unfortunately, 2 prior peritoneal biopsies from years prior were nondiagnostic in our patient.

Delay in diagnosis may be attributed to the need for a tissue diagnosis and the rarity of disease with only a few hundred cases have been described in the medical literature.^[4]^ Our case is particularly unusual due to it presentation as meningitis with hypoglycorrhachia. While neoplasm is within the standard differential considerations of hypoglycorrhachia, ECD has been described as presenting with meningitis with hypoglycorrhachia in only one other published case the authors are aware.^[9]^ In general, the nature of neurologic involvement ECD can be characterized as infiltrative (44%), meningeal (37%), or composite (19%) disease.^[10]^ Infiltrative disease involves parenchymal masses or lesions of the brain with meningeal pattern disease being dural thickening or meningioma-like tumors.^[10]^ The one case of ECD presenting as meningitis published had infiltrative disease with diagnosis by biopsy of a brain mass interestingly, and we are unaware of any cases of meningeal disease presenting clinically as meningitis.^[9,10]^ One case of leptomeningeal spinal disease on imaging is described, but literature review of cases with CSF analysis results published are notable for normal cell counts and glucose and only elevated protein levels in meningeal disease.^[10]^ Prior literature in fact recommends against lumbar puncture in ECD patients as ECD histocytes rarely appear in the CSF.^[11]^

Imaging in ECD most commonly reveals osteosclerotic bone lesions on plain films or bone scintigraphy, although osteolytic lesions may be present in 30% of patients.^[4]^ In addition to commonly involving the extraskeletal long bones, ECD bone lesions can be observed on imaging of the calvarium in over 40% of patients.^[7]^ For patients with pulmonary disease a restrictive pattern on pulmonary function tests can be found due to fibrosis disease, and periaortic fibrosis in cardiovascular involvement can appear as a “coated aorta” on CT.^[4]^ Imaging of ECD patients with parenchymal neurologic disease demonstrates enhancing lesions which can be nodular or infiltrative.^[7]^ Dural-based lesions in meningeal disease also enhance on imaging.^[7]^ PET-CT has been used to delineated areas of activity of ECD, with presence of the BRAF mutation correlated with PET avid disease..^[12]^ Our patient’s PET-CT demonstrated increased uptake only in the lung and spleen, with the spleen being described as an uncommon site of involvement in ECD.^[13]^

It is uncertain whether earlier diagnosis in our patient would have improved his outcome. Response to treatment has been improved with kinase inhibitor therapies targeting the mitogen-activating protein kinase pathway. Corticosteroids have been utilized, but these and other traditional therapies such as other immunosuppressants, interferon-alpha, and cytotoxic chemotherapy usually lead to only a partial or null response in published case series.^[7]^ Our case and the other published case of ECD induced meningitis both unfortunately died after steroid treatment.^[9]^ Overall prognosis of neurologic ECD can vary though, with mean survival of 9 years after diagnosis.^[7,10]^

## 4. Conclusion

Our case of hypoglycorrhachia from a neoplasm, ECD, is a rare presentation of a rare diagnosis, a true challenge for the diagnostician. ECD may present as meningitis though.

## Author contributions

C.P. drafted the initial article except for the pathology sections which were written by C.W. All authors contributed to conception, editing and revision or the article and care of the patient.

## References

[1] Chow E Troy SB. The differential diagnosis of hypoglychorrhachia in adult patients. Am J Med Sci. 2014;348:186–90. 24326618 10.1097/MAJ.0000000000000217 PMC4065645

[2] Anwar A Gudlavalleti A Ramadas P. Carcinomatous Meningitis. [Updated 2021 Nov 7]. Zaidi S, ed. In: StatPearls [Internet]. Treasure Island (FL): StatPearls Publishing, 2022. 32809651

[3] Wadayama T Shimizu M Kimura I. Erdheim-Chester disease involving the central nervous system with latent tuberculosis. Intern Med. 2022;61:2661–6. 35135916 10.2169/internalmedicine.8564-21 PMC9492489

[4] Mazor R Manevich-Mazor M Shoenfied Y. Erdheim-Chester disease: a comprehensive review of the literature. Orphanet J Rare Dis. 2013;8:137. 24011030 10.1186/1750-1172-8-137 PMC3849848

[5] Cavalli G Guglielmi B Berti A Campochiaro C Sabbadini MG Dagna L. The multifaceted clinical presentations and manifestations of Erdheim-Chester disease: comprehensive review of the literature and of 10 new cases. Ann Rheum Dis. 2013;72:1691–5. 23396641 10.1136/annrheumdis-2012-202542

[6] Pan Z Kleinschmidt-DeMasters BK. CNS Erdheim-Chester disease: a challenge to diagnosis. J Neuropathol Exp Neurol. 2017;76:986–96. 29096034 10.1093/jnen/nlx095

[7] Bhatia A Hatzoglou V Ulaner G. Neurologic and oncologic features of Erdheim-Chester disease: a 30-patient series. Neuro Oncol. 2020;22:979–92. 31950179 10.1093/neuonc/noaa008 PMC7339889

[8] Boyd LC O’Brien KJ Ozkaya N. Neurologic manifestations of Erdheim-Chester disease. Ann Clin Transl Neurol. 2020;7:497–506. 32227455 10.1002/acn3.51014 PMC7187721

[9] Northern ME Lee RM Katz JM Bergl PA Obeidat AZ. Clinical reasoning: a 42 year-old woman with mysterious monocytic meningitis. Neurology. 2021;97:449–54. 33947775 10.1212/WNL.0000000000012155

[10] Lachenal F Cotton F Desmurs-Clavel H. Neurologic manifestations and neuroradiological presentations of Erdheim-Chester disease: report of 6 cases and systemic review of the literature. J Neurol. 2006;253:1267–77. 17063320 10.1007/s00415-006-0160-9

[11] Globerman H Burstein S Girardina PJ Winchester P Frankel S. A xanthogranulomatous histiocytosis in a child presenting with short stature. Am J Pediatr Hematol Oncol. 1991;13:42–6. 1903027 10.1097/00043426-199121000-00010

[12] Young JR Johnson GB Murphy RC Go RS Broski SM. 18F-FDG PET/CT in Erdheim-Chester disease: imaging findings and potential BRAF mutation biomaker. J Nucl Med. 2018;59:774–9. 29097410 10.2967/jnumed.117.200741

[13] Stefano GD Granai M Giudici F Roselli G Lazzi S Santi R. Xanthamatous inflammatory infiltrate involving the spleen: an usual presentation of Erdheim-Chester disease and review of the literature. Am J Case Rep. 2021;22:e931060. 34083501 10.12659/AJCR.931060 PMC8183300

